# Noncoding RNA PVT1 in osteosarcoma: The roles of lncRNA PVT1 and circPVT1

**DOI:** 10.1038/s41420-022-01192-1

**Published:** 2022-11-15

**Authors:** Tingrui Wu, Ziyu Ji, Hao Lin, Bo Wei, Guohao Xie, Guangju Ji, Shijie Fu, Wenhua Huang, Huan Liu

**Affiliations:** 1 Orthopedic Center, Affiliated Hospital of Guangdong Medical University, 524001 Zhanjiang, China; 2 Department of Orthopedics, Affiliated Traditional Chinese Medicine Hospital, Southwest Medical University, 646000 Luzhou, China; 3 Guangdong Engineering Research Center for Translation of Medical 3D Printing Application, Guangdong Provincial Key Laboratory of Medical Biomechanics, National Key Discipline of Human Anatomy, School of Basic Medical Sciences, Southern Medical University, 510000 Guangzhou, China; 4 Guangdong Medical Innovation Platform for Translation of 3D Printing Application, The Third Affiliated Hospital of Southern Medical University, 510000 Guangzhou, China

**Keywords:** Bone cancer, Sarcoma

## Abstract

Osteosarcoma (OS) is the most common primary malignant bone tumor in children and teenagers and is characterized by high malignant potential, rapid disease progression and high disability and mortality rates. Recently, noncoding RNAs (ncRNAs) have attracted the attention of many scholars due to their major regulatory roles in gene expression. Among them, lncRNA PVT1 and circPVT1 encoded by the PVT1 gene have been the focus of many studies; they are upregulated in OS, and abundant evidence indicates that lncRNA PVT1 and circPVT1 play key roles in the occurrence and development of OS. This review summarizes the mechanisms of action of lncRNA PVT1 and circPVT1 in regulating apoptosis, proliferation, glycolysis, invasion, migration and epithelial–mesenchymal transition (EMT) in OS and discusses their clinical applications in diagnosis, prognosis determination and drug resistance treatment, with the aim of helping researchers better understand the regulatory roles of lncRNA PVT1 and circPVT1 in OS progression and providing a theoretical basis for the development of early screening and accurate targeted treatment strategies and prognostic biomarkers for OS based on lncRNA PVT1 and circPVT1.

## Facts

1. Osteosarcoma is the most common primary malignant bone tumor in children and adolescents, and its high malignancy and easy metastasis lead to a poor prognosis.
2. Both lncRNA PVT1 and circPVT1 are noncoding RNAs that upregulate expression in a variety of tumors and regulate the malignant biological behaviors of tumor cells.
3. LncRNA PVT1 and circPVT1 encoded by the PVT1 gene are upregulated in osteosarcoma and play key roles in the occurrence and development of osteosarcoma.

## Open questions

1. Are both lncRNA PVT1 and circPVT1 upregulated in the same osteosarcoma patient?
2. Do lncRNA PVT1 and circPVT1 have the same effects and mechanisms for regulating osteosarcoma?
3. Can lncRNA PVT1 and circPVT1 be clinically applied as diagnostic biomarkers and therapeutic targets?

## Introduction

In children and adolescents, osteosarcoma (OS) is the most common primary malignant bone tumor, and it places heavy physiological and economic burdens on patients [1]. OS most commonly occurs in the metaphysis of long bone and is characterized by the production of an osteoid matrix by transformed osteoblastic cells [2]. OS is a highly malignant disease, with ~15–20% of patients presenting at diagnosis with evidence of metastasis, mainly to the lungs, and the prognosis of patients with distant metastatic lesions is still very poor [3]. With great advances in surgical techniques and neoadjuvant chemotherapy, the mortality rate of OS patients has gradually decreased, and the prognosis has improved significantly [4]. However, the 5-year survival rate of OS patients with lung metastasis, tumor recurrence after comprehensive treatment, or multidrug resistance remains <20% [5]. Many basic and clinical studies have identified biomarkers related to OS, but there is a lack of effective biomarkers for accurately diagnosing early OS [6]. Therefore, there is a need to further explore OS-related biomarkers as reliable early diagnostic markers, prognostic indicators, and accurate therapeutic targets in clinical practice and to elucidate their possible effects and mechanisms to improve the current diagnosis and treatment of OS.

With rapid progress in the development of RNA-seq technology, many noncoding RNAs (ncRNAs) previously considered “transcription noise” have been screened and identified. These ncRNAs account for more than 98% of all human transcripts and are the main components of the transcriptome [7, 8]. According to the formation mechanism and structure, ncRNAs can be roughly divided into linear ncRNAs and circular RNAs (circRNAs). Linear ncRNAs can be divided into two major subtypes based on relative length; ncRNAs of <200 nucleotides (nt) are referred to as small or short ncRNAs, while those longer than 200 nucleotides are referred to as long noncoding RNAs (lncRNAs). Small ncRNAs can range in length from several nt to 200 nt, while lncRNAs can contain several thousand bases; microRNAs (miRNAs), which are ~20 nt long, are currently the smallest known type of ncRNA and have been extensively studied [9]. MiRNAs play important roles in almost all aspects of the malignant biological behavior of cancer; for example, they regulate the proliferation, apoptosis, invasion/metastasis, and angiogenesis of tumor cells and inhibit gene expression at the posttranscriptional level mainly by targeting complementary sequences in the 3’ untranslated region (UTR) of mRNA [10].

Most lncRNAs are transcribed by RNA polymerase II and may have cap structures and poly-A tails; lncRNAs are structurally similar to mRNAs but lack protein-coding capability [11]. LncRNAs mainly interact with DNA, mRNA, miRNA, and protein and regulate gene expression at multiple levels, including at the transcriptional, posttranscriptional, epigenetic, translational, and posttranslational levels [12]. LncRNAs have been found to play vital regulatory roles in various aspects of OS cell biological behavior, such as cell apoptosis, proliferation, invasion, differentiation, and autophagy [13, 14]. CircRNAs have attracted the attention of many scholars in the field of molecular biology. Unlike classical linear RNAs, circRNAs have a continuous, stable, covalently closed cyclic structure that lacks a 5’-cap and a 3’-poly(A) tail; circRNAs are therefore resistant to nucleic acid exonuclease or RNase R digestion. Most circRNAs have tissue-specific or developmental stage-specific expression and are highly abundant and conserved across different species [15, 16]. Therefore, circRNAs could be the most promising early diagnosis biomarkers and therapeutic targets. CircRNAs function as tumor suppressor genes or oncogenes in the malignant progression of OS by affecting processes including apoptosis, invasion, growth, differentiation, migration, drug resistance, and cachexia [17]. Recent studies have shown that various differentially expressed lncRNAs, such as TUG1, LOXL1-AS1, GAS5, NEAT1, HULC, and ANRIL, and circRNAs, such as circTADA2A, circFAT1, and circMYO10, are involved in the progression of OS [13, 18].

Many recent studies have shown that lncRNA PVT1 and circPTV1 are upregulated in a variety of tumors and play important regulatory roles in the malignant biological behavior of tumors. The data indicate that lncRNA PTV1 is upregulated in breast cancer cells and tissues and that knocking down lncRNA PVT1 inhibits the migration and proliferation of breast cancer cells and induces apoptosis. Interestingly, circPVT1 is also upregulated in cell lines and tissues of breast cancer; it promotes proliferation, invasion, and migration and inhibits apoptosis through the AGR2–HIF-1α axis mediated by miR-29a-3p; therefore, this circRNA functions as an oncogene in breast cancer [19, 20]. High expression of lncRNA PVT1 is positively correlated with clinical stage, lymph node metastasis, and distant metastasis in lung cancer patients. Patients with high levels of lncRNA PVT1 have a significantly lower overall survival rate than those with low levels, and lncRNA PVT1 may represent a new biomarker and possible therapeutic target for patients with lung cancer [21]. Lung cancer patients with high circPVT1 expression exhibit aggressive clinicopathological features and a poor prognosis, and knocking out circPVT1 can inhibit lung cancer cell proliferation and induce apoptosis; thus, circPVT1 acts as an oncogene in lung cancer [22]. There is increasing evidence that lncRNA PVT1 is involved in the proliferation and differentiation of digestive system tumors and also in epithelial–mesenchymal transition (EMT); lncRNA PVT1 has great potential to facilitate the diagnosis and treatment of digestive system cancers [23]. Similarly, circPVT1 is upregulated in hepatocellular carcinoma, gastric cancer, and colorectal cancer and is involved in the complex regulation of digestive system tumors at different levels [24–26]. In addition, the upregulation of both lncRNA PVT1 and circPVT1 is involved in the regulation of urinary system tumors [27, 28]. In conclusion, various studies have demonstrated that both lncRNA PVT1 and circPVT1 are crucial for tumor development. Although PVT1 has been found to be upregulated in human OS, its mechanism of action and clinical significance remain unclear. We will discuss the biological function and significance of PVT1 in OS in this review. PVT1 is a potentially useful target for the early diagnosis and treatment of OS.

## Structural characteristics of lncRNA PVT1 and circPVT1

**Fig. 1:**
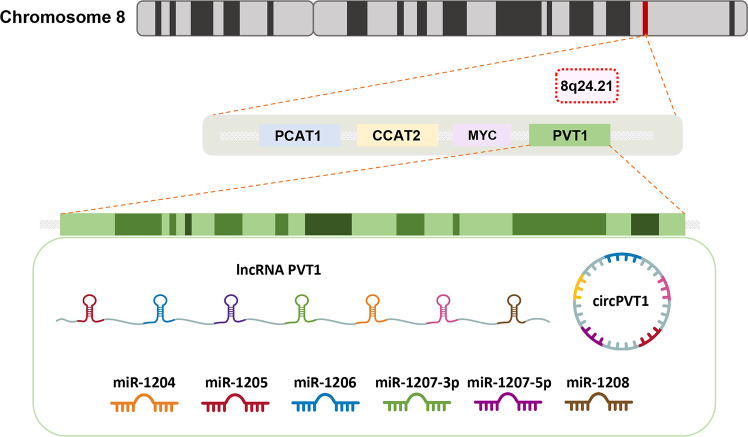
Structural characteristics of lncRNA PVT1 and circPTV1.

The human plasmacytoma variant translocation 1 (PVT1) gene is found on chromosome 8q24.21, near the MYC oncogene, and encodes both circPVT1 and lncRNA PVT1 (Fig. 1). Many studies have shown that the PVT1 genomic region is closely related to the malignant progression of tumors. The site of PVT1 was initially defined as hundreds of kilobases (kb) downstream of B-cell or T-cell lymphoma MYC, which harbors a set of chromosomal translocations or viral integration breakpoints [29]. Upregulated PVT1 can promote the expression of c-Myc by increasing its stability. In addition, PVT1 and c-Myc can interact to reciprocally regulate the expression and can synergistically promote the occurrence and development of tumors [30]. The PVT1 gene contains nine exons that can produce multiple transcripts by alternative splicing with lengths ranging from 2.7 to 3.3 kb; 26 isoforms of lncRNA PVT1 can be found in the NONCODE database (www.noncode.org) [31, 32]. CircPVT1 is derived from exon 2 of the PVT1 gene and is the product of reverse splicing and the formation of a closed-loop structure. The 26 isoforms of circPVT1 are labeled in the Circular RNA Interactome database (https://circinteractome.nia.nih.gov/index.html) [33, 34]. In addition to these splice variants, a set of six annotated miRNAs, including miR-1204, miR-1205, miR-1206, miR-1207-3p, miR-1207-5p, and miR-1208, can also be produced by splicing the PVT1 gene [35].

## Mechanisms by which lncRNA PVT1 and circPVT1 regulate the progression of osteosarcoma

### Regulation of cell proliferation and apoptosis

**Fig. 2:**
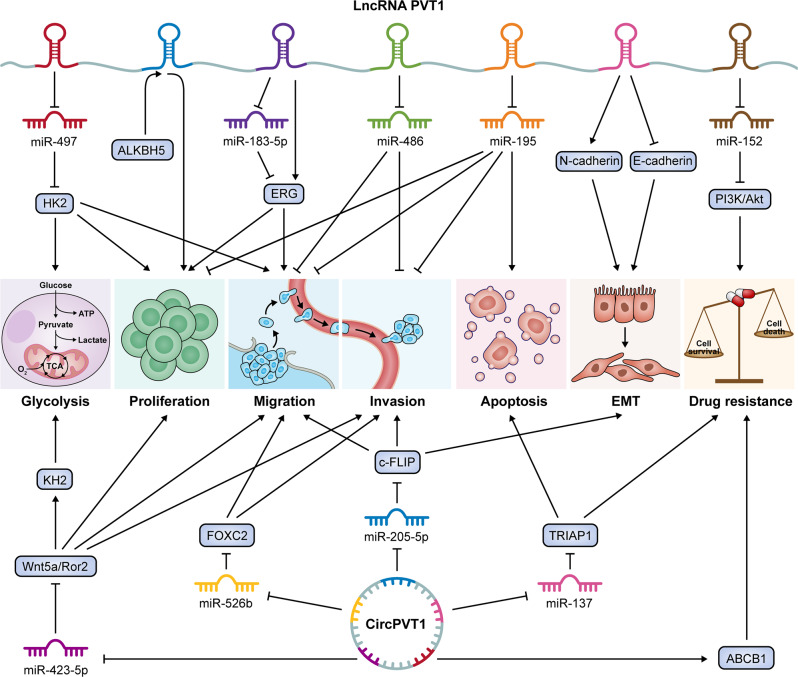
Mechanism by which lncRNA PVT1 and circPVT1 regulate the progression of osteosarcoma.

The regulation of cell proliferation is essential to maintain cell homeostasis, and disorder of this process is the main marker of cancer cells; proliferation is mainly controlled through the regulation of the cell cycle. Cyclin D1, a mitotic sensor and allosteric activator of CDK4/6, is one of the most common cell cycle regulators in cancer and is often overexpressed in cancer [36]. Overexpression of cyclin D1 leads to an imbalance in CDK activity, which leads to rapid cell growth under conditions of limited mitotic signals, thus bypassing key cellular checkpoints and eventually leading to tumor growth [37]. A previous study showed that cyclin D1 is upregulated in OS and can promote proliferation and inhibit apoptosis [38]. The cell cycle and apoptosis are inextricably linked, and cell proliferation and death must be dynamically regulated in cells to maintain tissue homeostasis. Apoptosis is a highly conserved programmed cell death pathway that is responsible for cell removal and steady-state maintenance during normal eukaryotic cell development. This pathway is regulated by the Bcl-2 protein family and determines the balance between cell survival and death [39]. Studies have found that when Bcl-2 and cyclin D1 are knocked down at the same time, the apoptosis of OS cells is significantly enhanced, and cell cycle arrest occurs; therefore, using lentiviral RNAi to simultaneously target Bcl-2 and cyclin D1 to regulate the proliferation and apoptosis of OS cells may be an effective therapeutic strategy [40]. Both circPVT1 and lncRNA PTV1 play important roles in promoting cell proliferation and inhibiting apoptosis in various tumors [41–45]. An earlier study revealed that the lncRNA PVT1 was upregulated in OS, and siRNA-mediated silencing of PVT1 inhibited the protein expression of BCL2 and CCND1 in OS cells, thereby inhibiting the proliferation of OS cells and promoting cell cycle arrest and apoptosis [46]. Another study found that circPVT1 levels were elevated in both OS tissues and cells, and knockout of circPVT1 significantly inhibited OS cell proliferation; moreover, knockout of circPVT1 in vivo significantly inhibited OS tumor growth and pulmonary metastasis [47]. According to these studies, PVT1 is a potential therapeutic target for OS and may control OS proliferation and apoptosis (Fig. 2).

### The role in epithelial–mesenchymal transition

Epithelial–mesenchymal transformation (EMT) is a process in which polarized epithelial cells lose their adhesion properties to obtain the functional phenotype of mesenchymal cells [48]. EMT is upregulated in almost all cancer types, and pertinent research has demonstrated that EMT is essential for the metastasis and invasion of cancer cells [49]. It is well known that one of the essential elements for inducing EMT is the downregulation of epithelial markers (such as E-cadherin) and the upregulation of mesenchymal markers (such as N-cadherin and vimentin). This process is also widely involved in regulating the progression of OS. For example, we found that after silencing CEACAM6 in OS cells, E-cadherin expression increased while N-cadherin and vimentin expression decreased, indicating that CEACAM6 regulates EMT to promote the metastatic characteristics of OS cells [50]. LncRNAs and circRNAs have been shown to have the ability to regulate EMT and therefore to be involved in the malignant biological behaviors of tumor cell metastasis and invasion. A study showed increased expression of 54 lncRNA species in colorectal cancer samples compared to normal colorectal epithelium samples; this increase in expression was generally associated with poor survival, and the effects were mediated by EMT-related genes [51]. Through EMT-related signaling pathways, EMT-related transcription factors, and other mechanisms, circRNAs can control the progression of EMT [52]. LncRNA PVT1 and circPVT1 have also been shown in numerous studies to be key modulators of EMT in OS. According to Xun et al., the expression of lncRNA PVT1 was increased in both OS tissues and cell lines and was closely associated with clinical features of OS, such as tumor differentiation, TNM stage, and distant metastasis. Further research revealed that knocking out the lncRNA PVT1 in OS cells could reduce vimentin and N-cadherin expression while increasing E-cadherin expression, thus preliminarily confirming that lncRNA PVT1 promotes the occurrence of OS by regulating EMT [53]. Liu et al. demonstrated that circPVT1 can serve as a “molecular sponge” to adsorb miR-205-5p, thereby promoting the expression of c-FLIP and enhancing EMT in vitro to induce OS invasion and metastasis [54]. Targeted regulation of EMT is currently considered a promising strategy for inhibiting tumor invasion and metastasis and improving the survival of cancer patients. However, the specific mechanism by which EMT is regulated has not been clarified, especially in OS, so it is worthy of further study (Fig. 2).

### Regulation of invasion and migration

Metastasis is one of the most prevalent causes of death associated with malignant tumors and is the most important factor in the poor treatment response and poor prognosis of OS [3, 55]. At present, there is sufficient clinical evidence that lncRNA PTV1 and circPTV1 are closely related to the metastasis of OS. However, research on the invasion and metastasis of OS has involved only in vitro Transwell and wound healing assays or in vivo subcutaneous tumorigenesis experiments and lung metastasis models. Data from multiple clinical studies indicate that high expression of lncRNA PVT1 in OS tissues compared to paracancerous tissues is positively associated with lymph node metastasis and distant metastasis [53, 56–58]. In contrast to primary OS tissues, metastatic OS tissues exhibit higher expression of the lncRNA PVT1 [46]. Invasion and metastasis are multistep and multilevel regulatory processes, and lncRNA PVT1 can regulate the complex process of tumor metastasis through miRNA [59]. Based on the competitive endogenous RNA (ceRNA) mechanism, lncRNA PVT1 sponges miR-484 and miR-195 and promotes the invasion and metastasis of OS [46, 56]. Similarly, circPVT1 competitively binds to miR-205-5p, miR-423-5p, and miR-526b via the same mechanisms to influence invasion and metastasis [47, 54, 58]. Another interesting study showed that BMSC-derived exosomes contain PVT1, and exosomes can transport PVT1 to OS cells and promote the growth and metastasis of OS cells [60]. These findings shed light on the mechanism by which lncRNA PTV1 and circPVT1 influence OS metastasis (Fig. 2).

### Regulation of the glycolytic pathway

Energy metabolism changes are biochemical fingerprints of cancer cells and represent one of the hallmarks of cancer [61]. Metabolism differs greatly between cancer cells and normal histiocytes. Tumor cells have a high proliferation rate and strong metabolic activity and can resist cell death pathways such as apoptosis [62]. In the 1920s, German scientist Warburg pointed out that cancer cells showed more glycolytic activity than normal cells. Even under aerobic conditions, cancer cells tend to metabolize glucose into lactic acid (aerobic glycolysis), a process now known as the Warburg effect [63]. The intrinsic mechanism of the Warburg effect is very complex and is related to oncogene activation, tumor suppressor gene inactivation, abnormal expression of sugar metabolizing enzymes, changes in the tumor microenvironment, and other factors and is closely related to the occurrence and development of tumors. Glucose uptake by tumor cells is very high because tumor cells grow and proliferate very rapidly, and this process is accompanied by the production and accumulation of large amounts of lactic acid and the upregulation of the key regulatory factor HK2, in accordance with the Warburg effect [64, 65]. There is increasing research evidence that lncRNAs and circRNAs can control tumor cell metabolism by affecting the levels of glycolysis-related enzymes, the expression of transcription factors (TFs), and the activation of signaling pathways [66, 67]. According to research by Song and colleagues, OS cells and tissues have higher levels of lncRNA PVT1 expression than the corresponding controls. The upregulation of PVT1 results in increased glucose uptake, massive accumulation of lactic acid, and increased expression of HK2 in cells. The recent finding that lncRNA PVT1 promotes OS cell glycolysis, proliferation, and migration mainly through the miR-497/HK2 pathway highlights the relationship between lncRNA and OS cell glycolysis [68]. Interestingly, circPVT1 can also regulate the progression of OS through the glycolytic pathway. By targeting the regulation of Wnt5a and Ror2 expression, circPVT1 is increased in both OS tissues and cell lines and inhibits the glycolysis, as well as the proliferation, invasion, and migration, of OS cells [47]. Therefore, lncRNA PVT1- or circPVT1-mediated glycolysis may be an effective target for OS treatment (Fig. 2).

## Diagnostic and prognostic biomarkers

Although there is increasing evidence that lncRNA PVT1 and circPTV1 may be biomarkers for the diagnosis and prognosis evaluation of several common malignancies, integrated analyses in OS are rare [69–72]. LncRNA PVT1 and circPVT1, as prognostic and diagnostic biomarkers of OS, have been repeatedly identified in multiple studies (Table 1). Despite recent significant advances in the diagnosis and treatment of OS, the quality of life and overall survival of affected patients remain unsatisfactory. The main causes of a poor prognosis in OS patients may be metastasis, frequent tumor recurrence, and drug resistance, which are closely related to delayed diagnosis [73]. Therefore, there is an urgent need to improve the early diagnosis approaches for OS so that patients can receive timely treatment and improve their prognosis. The expression of lncRNA PVT1 and circPVT1 is upregulated in a variety of cancers; these molecules can be detected in serum, and their clinical application potential as diagnostic biomarkers has been predicted and verified in many studies [57, 74, 75]. Compared to those in nonmelanoma controls, the serum levels of lncRNA PVT1 in melanoma patients are significantly higher; this marker has a sensitivity of 94.12%, a specificity of 85.11%, and an area under the ROC curve (AUC) of 0.9387 for distinguishing between patients with melanoma and controls; this finding suggests that serum lncRNA PVT1 may be a new biomarker for the early diagnosis of melanoma [76]. Because circRNAs have a more stable covalent closed-loop structure than lncRNAs, they are less likely to be degraded in vivo and therefore are more likely to be detected in bodily fluids. Compared with the healthy control group, patients with benign bone tumors and OS showed a gradual increase in the plasma levels of circPVT1, and ROC survival curve analysis showed that circPVT1 had higher diagnostic efficiency and higher sensitivity and specificity than the commonly used clinical biomarker alkaline phosphatase (ALP) [57]. To date, there have been few reports on lncRNA PVT1 as a diagnostic biomarker of OS, and further research on this topic is needed.

**Table 1:** LncRNA PVT1 and circPVT1 as prognostic and diagnostic biomarkers of osteosarcoma.

LncRNA/circRNA	Number of patients/controls	Clinical stage	Diagnostic properties	Prognostic properties	References
		IIB/III, *p*-value	AUC, *p*-value	*p*-value	
lncRNA PVT1	48/48(tissue)	IIB/III, 0.008	–	0.0001	Yan, M et al. 2020 [56]
lncRNA PVT1	70/70(tissue)	IIB/III, 0.008	–	0.0087	Chen, Shuo et al. 2020 [77]
lncRNA PVT1	46/46(tissue)	III, <0.001	–	0.047	Song, Jingyi et al. 2017 [68]
lncRNA PVT1	78/78(tissue)	III, <0.001	–	<0.001	Xun, Chuanhui et al. 2021 [53]
lncRNA PVT1	26/26(tissue)	–	–	<0.05	Zhou, Quan et al. 2016 [46]
CircPVT1	80/80(tissue) and 50/20(serum)	–	0.871, <0.001	0.002	Kun-Peng, Zhu et al. 2018 [57]
CircPVT1	48/48(tissue)	IIB/III, 0.008	–	0.0053	Yan, Ming et al. 2020 [58]

At present, tumor recurrence and metastasis represent difficulties in the treatment of OS and are key factors affecting prognosis. Obtaining a more accurate prognosis is of great significance for the design and selection of treatment options. Studies have revealed a close connection between circPVT1 and lncRNA PVT1 and the pathological and clinical characteristics of OS and can be used as important biomarkers for evaluating the prognosis of OS. Kaplan‒Meier survival analysis revealed that the lncRNAs PVT1 and circPVT1 were highly expressed in OS and were negatively correlated with survival rate, indicating the potential of these molecules as prognostic biomarkers of OS [46, 53, 56–58, 68, 77]. Further analysis of the above studies revealed the significant and interesting phenomenon that patients with high expression of lncRNA PVT1 and circPVT1 are most likely to have stage IIB/III OS and least likely to have stage I/IIA OS [53, 56, 58, 68, 77]. The high expression of lncRNA PVT1 and circPVT1 in OS is consistent with trends in survival and clinical staging.

## Resistance to therapeutic agents

Due to the clinical application of adjuvant chemotherapy and neoadjuvant chemotherapy, the survival rate of OS patients with only primary local lesions has significantly improved [78]. Numerous clinical research projects have demonstrated that methotrexate, doxorubicin, cisplatin, and ifosfamide are currently the most effective drugs against OS, but the optimal drug combination and dose are still controversial [79–81]. Gemcitabine as second-line therapy shows satisfactory antitumor activity and safety in patients with recurrent or refractory OS after standard chemotherapy [82–84]. Since the 1970s, the 5-year survival rate of OS patients with local lesions has remained at ~75%, while the 5-year survival rate of OS patients who experience chemoresistance or have distant metastasis has dramatically decreased to 20%. One of the leading causes of OS treatment failure is drug resistance [85]. Therefore, further research into the molecular mechanisms underlying resistance to conventional chemotherapy is required to accurately intervene to target chemoresistance and improve sensitivity to chemotherapy drugs by formulating new and more effective treatment strategies to improve the survival rate. Drugs can leave cells when multidrug-resistant ABC transporters such as multidrug-resistant protein 1 (MRP1/ABCC1) and P-glycoprotein (P-gp/MDR1/ABCB1) are overexpressed, a process that may be an important cause of chemoresistance [86–88]. There is increasing evidence that a variety of ncRNAs, including miRNAs, lncRNAs, and circRNAs, play key roles in the progression of OS and modulate chemosensitivity through various mechanisms [89–91]. Sun et al. found that lncRNA PVT1 could participate in the chemoresistance of OS cells by activating the c-MET/PI3K/AKT signaling pathway [92]. In addition, Peng et al. suggested that knocking out circPVT1 in vitro can partially reverse the resistance of OS cells to doxorubicin and cisplatin by reducing the expression of ABCB1 [57]. Another study showed that knocking out circPVT1 can downregulate the levels of ABCB1 and MRP-1 in doxorubicin-resistant OS cells, which also indicates that silencing circPVT1 may reduce the resistance of OS cells to doxorubicin, and the drug resistance effect of circPVT1 has also been verified in vivo in OS cells [93]. LncRNA PVT1 and circPVT1 have the potential to be used as biomarkers in the treatment of OS, potentially improving the efficacy of current therapeutic regimens and serving as potential targets for the development of novel therapeutic interventions (Fig. 2).

## Conclusions and future perspectives

**Fig. 3:**
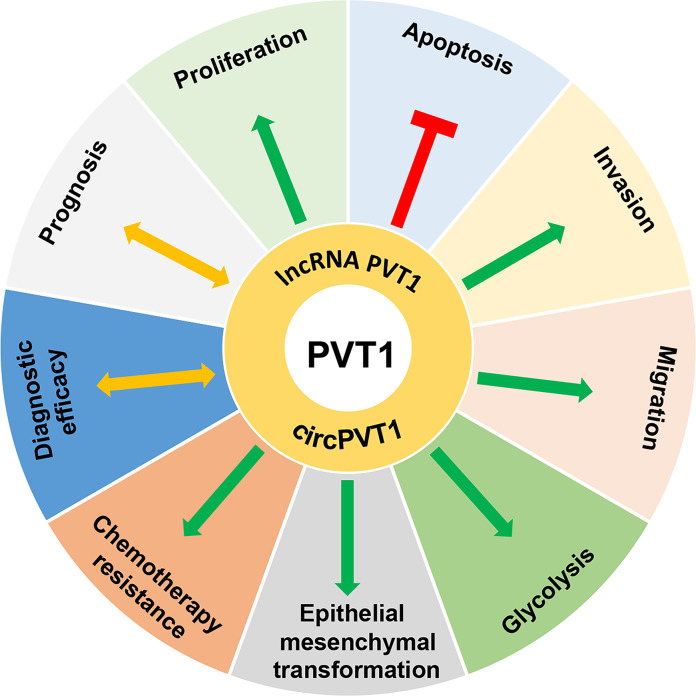
The role of lncRNA PVT1 and circPVT1 in the progression of osteosarcoma. Green connections indicate promotion, red connections indicate suppression, and yellow connections indicate the presence of correlation.

This paper summarizes the rich and multidisciplinary research progress regarding two ncRNAs derived from the PVT1 gene, lncRNA PVT1, and circPVT1, in OS. A large number of OS-related studies have identified a trend of upregulated expression of lncRNA PVT1 and circPVT1, and in other studies, both lncRNA PVT1 and circPVT1 have been found to affect malignant biological behaviors such as the proliferation, apoptosis, invasion, and migration of OS cells. In terms of clinical application, lncRNA PVT1 and circPVT1 have shown good efficacy in diagnosis and prognosis and positive application prospects in relevant aspects of treatment resistance; there is preliminary evidence that these molecules will potentially become useful biomarkers that can be used to diagnose and treat OS (Fig. 3). In conclusion, these advances may help establish personalized and precise treatment for OS and improve the prognosis of patients.

While the results with regard to lncRNA PVT1 and circPVT1 in basic studies on OS are encouraging, further studies are needed; in particular, a multicenter independent cohort study is needed to verify the clinical value of lncRNA PVT1 and circPVT1. The current reality is that the translation of basic research to clinical research is rather difficult because although in vitro experimental models are easy to characterize in the lab, the correlation of these models with clinical practice is limited. In vivo models have greater clinical relevance but are limited by medical ethics, cost, and challenges in implementation. Since lncRNA PVT1 and circPVT1 are not highly conserved in animals, assessing the functional and mechanistic roles of lncRNA PVT1 and circPVT1 remains a significant challenge. Therefore, more research on the regulatory mechanisms of lncRNA PVT1 and circPVT1 in OS is needed to realize their potential for clinical application.

## Data Availability

All the data used to support the findings of this study are available in the paper.
